# Mixed Grazing and Dietary Supplementation Improve the Response to Gastrointestinal Nematode Parasitism and Production Performances of Goats

**DOI:** 10.3389/fvets.2021.628686

**Published:** 2021-04-23

**Authors:** Jean-Christophe Bambou, Willy Ceï, Rémy Arquet, Valériuse Calif, Bruno Bocage, Nathalie Mandonnet, Gisèle Alexandre

**Affiliations:** ^1^URZ, Unité de Recherches Zootechniques INRAE, Petit-Bourg, France; ^2^PTEA, Plateforme Tropicale d'Expérimentation sur l'Animal INRAE, Le Moule, France

**Keywords:** grazing system, nutrition, goats, gastrointestinal parasitism, production performance

## Abstract

Small ruminants are very affected by gastrointestinal nematode (GIN) parasitism worldwide. The improvement of the host protective response and the reduction of the probability of contact between hosts and parasites appear as very promising strategies. The objective of this study was to evaluate the effect of a combination of two components of these two strategies on natural GIN infection and production performances of Creole goat kids: dietary supplementation and a rotational mixed grazing system. One hundred and twenty weaned Creole kids and six Creole heifers were divided into four experimental batches: Splus (supplemented) vs. Szero (non-supplemented) × Mixed grazing (kids associated with heifers) vs. Control (kids alone), and grazing plots of forage grasses were balanced for live weight (LW) in both species. The feed intake, blood, and parasitological parameters and production performances between 3 and 11 months of age were monitored. The fecal egg count (FEC) was significantly lower and the packed cell volume (PCV) significantly higher in the Mixed grazing groups. No effect of supplementation was observed for FEC. In contrast, PCV, body condition score, and live weight were significantly higher in supplemented animals whatever the groups. Mixed grazing system and supplementation had significant effects on the slaughter parameters (*P* < 0.05), but there was no significant interaction. Our results suggested that the advantage driven either by mixed grazing or dietary supplementation on kids' performances would be equivalent, and the combination of both would improve the animal performances.

## Introduction

Small ruminants, especially goats, are very affected by gastrointestinal nematode (GIN) parasitism, a major pathology affecting tropical livestock farms (1). For decades, anthelmintic drugs were the main control strategy used by farmers, but the rise in anthelmintic resistance, their potential environmental side effects, and the legitimate societal demands for chemical residue-free animal products increase the need to develop additional and sustainable control strategies (2). These strategies are developed on two axes. The first aims at enhancing the host immune response by the exploitation of genetic resistance, nutritional supplementation, and potentially vaccination (3–5). The second is the reduction of the probability of contact between the parasite and the host by taking into account the life cycle of GIN to manage the pasture utilization (6).

Today, global schemes of parasitism management integrating a parsimonious use of classical practices and the different alternative control strategies have to be developed to improve the health, the welfare, and thus the production of pasture-based ruminants. The objective is no longer to control parasites by the use of only one control strategy, but rather to design biotechnical innovations, which aim to reach a favorable equilibrium for animal production. Thus, the risk of parasite evolution toward increased resistance to anthelmintic and virulence should decrease. This new emerging paradigm is in line with an agroecological approach for the conception of sustainable livestock systems to achieve an efficient agriculture conciliating food security and environmental protection (7, 8).

Mixed grazing is an integrated approach based on the relative host specificity of GIN species and the different behaviors of grazing ruminants (9, 10). The majority of studies on mixed grazing either concomitant or alternative, concern the association between sheep and cattle (11). Despite the importance of goat production for the supply of meat and milk in developing countries, a few studies have been carried out on mixed systems involving goats (11–13). In addition, the importance of dietary supplementation, especially protein supplementation, showed in numerous studies on resistance and resilience of sheep and goats to GIN infections, has been recently confirmed in two meta-analyses (14, 15). To our knowledge, the synergism between the nutritional and the pasture management strategies has never been investigated experimentally. Thus, the objective of our study was to evaluate the effect of a combination of feed supplementation and mixed grazing on the parasitological responses and the production performances including the carcass characteristics of a herd of goats in post-weaning fattening.

## Materials and Methods

### Ethics Approval and Consent to Participate

All measurements and observations on animals were carried out in accordance with the current law on animal experimentation and ethics, and approved by the French Ministry of Agriculture (authorization number: HC-69-2014-1) after evaluation by the Animal Care and Use Committee of French West Indies and Guyana (Comité d'Ethique en Matière d'Expérimentation Animale des Antilles et de la Guyane, C2EA-69).

### Animals, Management, and Experimental Design

This experiment was conducted during 8 months at the INRAE PTEA experimental farm in Guadeloupe (16° 20′ North latitude, 61° 30′ West longitude). All the animals owned by the INRAE PTEA are reared in this experimental farm since 1980. A total of 120 weaned male Creole kids (11.5 ± 1.62 kg live weight; 95 ± 6 days of age) and six Creole heifers (237 ± 16.4 kg; 381 ± 16 days of age) were used in a 2 × 2 experimental design to evaluate the effects of mixed grazing with cattle and diet supplementation on the feed intake, blood, and parasitological parameters and production performance of post-weaning kids between 3 and 11 months of age. The commercial concentrate (1.15 UFL and 150 g PDIN per kg DM) was composed of maize (68%), soybean cake (15%), wheat bran (11%), vitamin and mineral supplement (5%), and urea (1%).

The animals were randomly divided into four experimental groups (*n* = 30 kids/group and *n* = 3 heifers/mixed grazing group): Splus (supplemented) vs. Szero (non-supplemented) × Mixed grazing vs. Control; grazing plots of forage grasses were balanced for live weight in both species. The cattle were not included in the analyses. The kids were naturally infected while grazing. During the course of the experiment, an oral drenching with Levamisole (Polystrongle®, Merial, Lyon, France, 7.5 mg/kg Live Weight, LW) and Ivermectine (Oramec®, Merial, Lyon, France, 0.2 mg/kg LW) was performed for the animals showing packed cell volume (PCV) values below 15%, and the experiment was discontinued for them. No further measures were performed for these animals.

The animals were led on plots composed of a mixture of tropical grasses, following a five-plot rotation system, with a 7-day length of grazing per plot (i.e., a 28-day interval of grass regrowth). The animals (kids and heifers) of the two Mixed grazing plots were distributed over a 2.36-ha pasture divided into five plots of 0.472 ha each, and the kids of the Control group were distributed over a 0.78-ha pasture divided into five plots of 0.156 ha each. For each group, the load was 871 kg of LW/ha.

Energy and protein supplement in the form of commercial pellets (96.4% of dry matter, 93.5% of organic matter, 17% of crude protein, 13.3 neutral detergent fiber, 3.6% acid detergent fiber, and 0.3% acid detergent lignin) was distributed daily to the kids in the Mixed Grazing Splus- and Control Splus-supplemented groups at a rate of 20 g/kg LW^0.75^/day for the duration of the experiment. This level of supplementation was determined to be optimal for growth performance and carcass quality (16). The animals have free access to mineral and vitamin supplement in the form of lickstones and fresh water.

### Sampling and Methods

#### Forage Variables

Biomass and chemical composition of the herbage availability were measured for each plot at the entrance and the exit of the animals. Measurements of average grass height over an area of 0.09 m^2^ each were representatively made on the “Control” and “Mixed” plots, with 30 and 90 points, respectively. Ten and 15 forage samples representative of the “Control” and “Mixed” plots were taken using a portable mower over an area of 0.09 m^2^, respectively. The forage samples were analyzed by the following AFNOR methods: dry matter (DM, AFNOR NF V18-109), ash (AFNOR NF V18-101), and total crude protein content (CP, N × 6.25, AFNOR NF V18-120). Plant wall constituents, acid detergent fiber (ADF), acid detergent lignin (ADL), and neutral detergents (NDF) were determined according to the Van Soest method (17).

#### Parasitological Variables

Individual blood and fecal samples were taken monthly from each animal in the same order from 07.00 to 09.00 h a.m. Approximately 10 g of feces was taken from each animal and placed in sterile plastic tubes. These samples were used to determine the fecal excretion of GIN eggs [fecal egg count, expressed as eggs per gram of feces (FEC)] by the modified McMaster method using a saturated NaCl solution and a centrifugation at 1,700 g (18).

Blood samples were collected after feces sampling in EDTA tubes (Becton Dickinson, Plymouth, UK) to measure the packed cell volume (PCV) by using the microhematocrit capillary method.

#### Animal Performances

The kids were weighed every month just after feces and blood sampling. Their fattening condition was assessed monthly with a body condition score (BCS) ranging from 1 (lean) to 5 (fat) by two skilled technicians. The individual live weight was used to estimate the weight at fixed age: 95 days (LW 95), 185 days (LW 185), and 275 days (LW 275). The average daily weight gain (ADG) between 95 and 185 days (ADG 95–185), 185 and 275 days (ADG 185–275), and 95 and 275 days (ADG 95–275) was calculated. Heifers were weighed monthly in the same order to adjust the stocking rate.

#### Carcass Characteristics

Sentinel kids (six per group) were slaughtered at 12 months of age. They were representative of their group in terms of ADG. The unslaughtered animals were reintroduced in the flocks of the experimental farm for the flock renewal. The measurement methods used in this study are described in detail in previous research on the Creole goat carcass (19).

The animals were weighed on the day before and on the day of slaughter (24 h of fasting before slaughter). The animals were humanely slaughtered with a captive bolt pistol, which hits them on the head producing immediate unconsciousness following by exsanguination. Thereafter, the entire digestive tract was removed, weighed full, and then separated by compartment, emptied, and weighed. The peritoneal and mesenteric fats were removed and weighed. The weights of the head, feet, skin, liver, heart, trachea, lungs, and waste were recorded. The carcasses were weighed 1 h after slaughter [hot carcass weight (HCW)] and after 24 h of cooling to 4°C [cold carcass weight (CCW)].

The carcasses were graded (from 1 to 5) according to their conformation and the quantities of internal and external fat on the basis of a grid dedicated to sheep already adapted to Creole goat carcasses (19).

### Calculation and Statistical Analysis

For the pasture characteristics, the statistical analyses were performed using the statistical software R (R_Development_Core_Team, v 3.6.0) to compare the effects of goat/cattle association, supplementation, and their interaction. The analysis procedure was entirely described in detail previously (20).

The FEC, PCV, weight, and body condition score variables were analyzed using a linear mixed PROC MIXED model of SAS (Version 9, SAS Inst., Inc., Cary, NC, 1999). The FEC were logarithm transformed (ln (FEC + 15) to normalize residual variances. The ADG and PCV were studied with the initial live weight and initial PCV as covariate kept in the model when significant, respectively. Results are presented as least square means. Only the variables prior to the anthelmintic treatment were considered for the animals excluded from the experiment.

Carcass characteristics, except for those calculated as a proportion of the slaughter weight, were studied with the CCW used as the covariate and retained in the model when significant (*P* < 0.05).

## Results

The results on the pasture characteristics are presented in Table 1. The animal stocking rate at entry expressed in kg of LW per hectare (871 kg LW/ha) was balanced for both grazing systems. The forage biomass available at the entry into the paddock of the Mixed grazing system was significantly higher (*P* < 0.001) than that obtained in the Control system (Table 1). The dry matter (DM), organic matter (OM), and fiber (NDF, ADF, and ADL) rates were similar regardless of the management. Only the crude protein content (CP) rates was significantly higher in the Mixed grazing system (*P* < 0.05) for the plots at the entrance of the animals. There was no significant difference between the two grazing systems for the variables measured at the exit of the plots.

**Table 1:** Means of herbage characteristics in rotationally grazed pasture by growing Creole kids according to experimental groups.

**Pasture management**	**Control groups^a^**	**Mixed grazing groups^b^**	***P*-value^c^**
**At the entrance in the paddock**			
Biomass (g ha^−1^)	5,609	6,742	0.001
Dry matter content (%)	30.4	29.7	NS
Organic matter^d^	900	895	NS
Crude protein^d^	81	96	0.05
Neutral detergent fiber^d^	713	685	NS
Acid detergent fiber^d^	361	335	NS
Acid detergent lignin^d^	58	53	NS
**At the exit of the paddock**			
Biomass (g.ha^−1^)	4,649	4,782	NS
Dry matter content (%)	31.3	31.8	NS
Organic matter^d^	906	899	NS
Crude protein^d^	73	74	NS
Neutral detergent fiber^d^	724	673	NS
Acid detergent fiber^d^	365	323	NS
Acid detergent lignin^d^	70	70	NS

a *Control groups: kids reared alone (supplemented and non-supplemented)*.

b *Mixed grazing groups: kids reared with heifers (supplemented and non-supplemented)*.

c *Effect of pasture management (PM): level of significance; NS: nonsignificant*.

d *Composition in g.kg^–1^ DM^–1^*.

**Figure 1:**
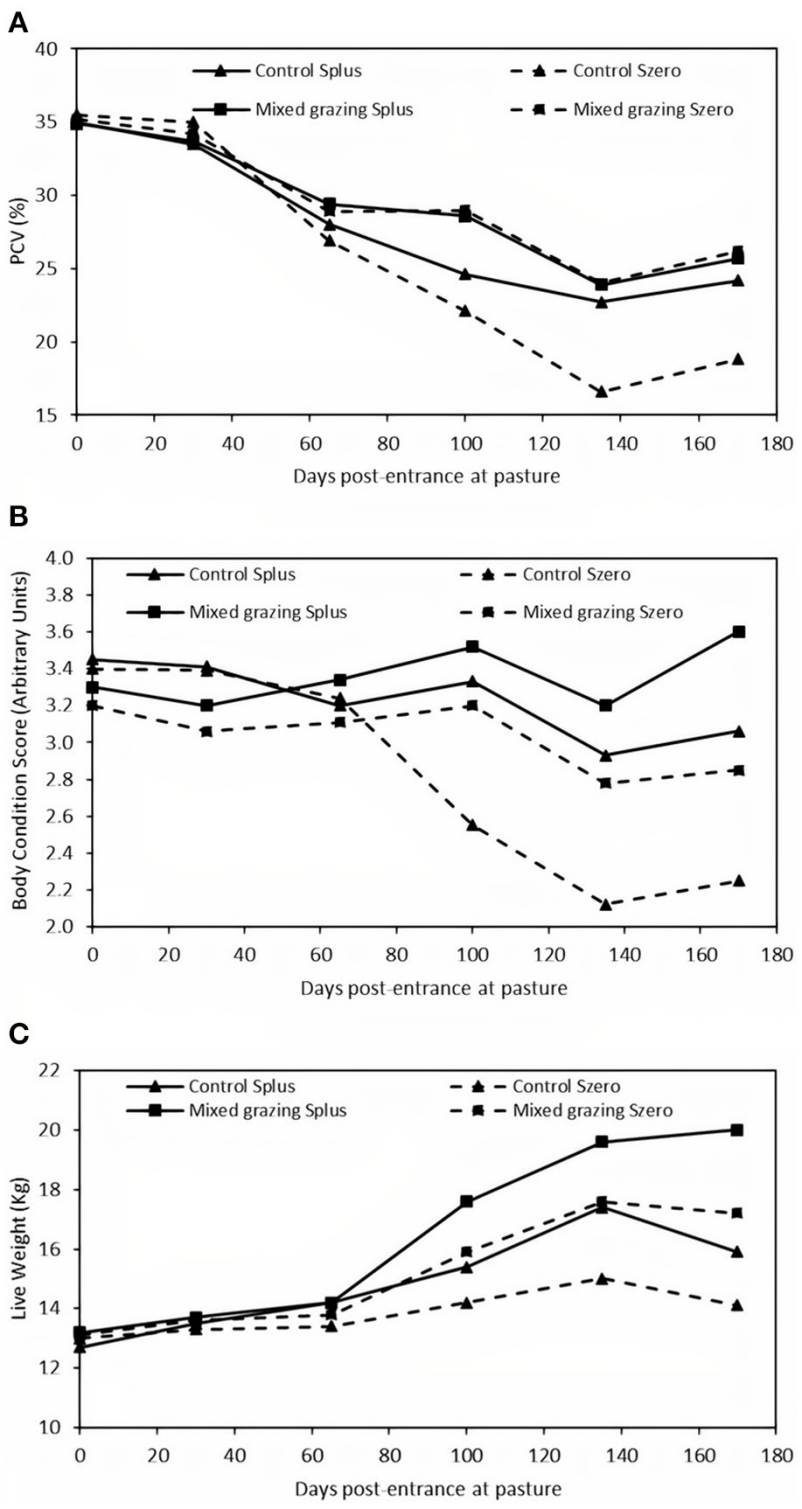
Least square means of packed cell volume (PCV) **(A)**, body condition score (BCS) **(B)**, and live weights (LW) **(C)** of kids according to the association and the supplementation levels (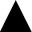 Control and 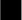 Mixed grazing). For all means, the solid (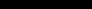) and hatched (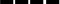) lines represent, respectively, the mean values for supplemented and non-supplemented kids.

**Figure 2:**
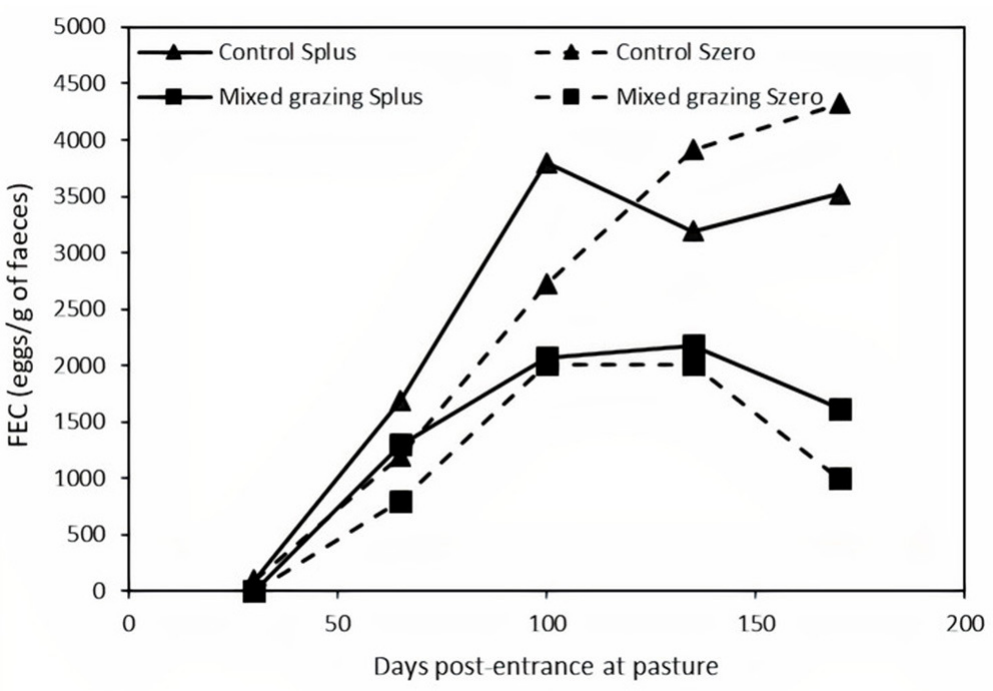
Least square means of fecal egg count (FEC) of kids according to the association and the supplementation levels (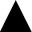 Control and 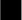 Mixed grazing). For all means, the solid (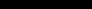) and hatched (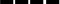) lines represent, respectively, the mean values for supplemented and non-supplemented kids.

Grazing management and supplementation had a significant effect on the PCV (*P* < 0.05, Figure 1A). The PCV of supplemented kids in the mixed grazing system (Mixed grazing Splus) was significantly higher than that of the control non-supplemented kids (Control Szero). Within the Control groups, the PCV of supplemented kids was higher. PCV values decreased over time, but the ranking between groups remained similar. Mixed grazing Splus kids had higher values of PCV compared with the Control Szero ones, which decreased to 18% after 6 months of grazing. The number of anthelmintic-treated animals for which the experiment was discontinued is presented in Table 2. The experiment was discontinued for a total of 25 animals initially allocated to Control Splus and Szero groups (*n* = 16 and 5, respectively) and Mixed grazing Splus and Szero groups (*n* = 3 and 1, respectively). FEC increased significantly whatever the groups until 100 days post-entrance at pasture (Figure 2). From 100 to 170 days post-entrance at pasture, FEC decreased significantly for the Mixed grazing kids (*P* < 0.05) but not for the Control ones. Control kids had least square means of FEC higher than Mixed grazing kids (*P* < 0.05), but no effect of supplementation and no interaction between supplementation and grazing system was observed.

**Table 2:** Number of animals anthelmintic treated and least square means of fecal egg count according to the experimental groups.

**Days post-entrance at pasture^1^**	**Control groups^2^**		**Mixed grazing groups^3^**	
	**Szero**	**Splus**	**Szero**	**Splus**
30	0	0	0	0
65	0	2	0	0
100	2	0	1	0
135	8	0	2	1
170	6	3	0	0
Fecal egg count^4^ (eggs/g of feces)	1,603 ± 504^a^	1,683 ± 567^a^	815 ± 287^b^	839 ± 274^b^

*LS means with different superscripts are significantly different (P < 0.05)*.

1 *Days post-entrance at pasture: At each day, the packed cell volume was measured, and when the values were below 15, the animals were treated with anthelmintics, and the experiment was discontinued for them*.

2 *Control groups: kids reared alone (supplemented and non-supplemented)*.

3 *Mixed grazing groups: kids reared with heifers (supplemented and non-supplemented)*.

4 *Fecal egg count: The FEC was measured at each indicated days post-entrance. The values are the least square means of the 5 time points*.

The BCS decreased over time except for the Mixed Splus group (*P* < 0.05, Figure 1B). This decrease was more pronounced from 100 days post entrance at pasture. In contrast, the BCS of the Mixed grazing Splus group increased slightly but significantly from 3.2 to reach 3.6 points of BCS at 170 days post-entrance at pasture (*P* < 0.05).

The evolution of the least square means of LW was different among groups, but no significant interaction between grazing management and supplementation was observed (Figure 1C). During the first months (between 30 and 100 days post-entrance at pasture), the Mixed grazing Splus group had a higher LW than the Control Szero group (*P* < 0.05), and there was no difference between the Mixed grazing Szero and the Control Splus groups. Thereafter, the kids of the Mixed grazing Splus group showed the highest LW (*P* < 0.01). No difference was observed between the kids from the Mixed grazing Szero and the Control Splus groups until 170 days when the Mixed grazing Szero had higher LW (*P* < 0.01). From 100 until 170 days post-entrance at pasture, the kids of the Control Szero group had the lowest LW.

There was no significant interaction between the factors analyzed for the slaughter parameters (i.e., slaughter live weight and empty live weight, hot and cold carcass weight, and carcass yield) (Table 3). Grazing system and supplementation had significant effects on the slaughter parameters (*P* < 0.05). However, commercial carcass yield did not change. Carcass conformation, internal fat, and external fat scores increased, although no significant effect of grazing system and supplementation was statistically demonstrated. The proportions of abdominal fat increased with a significant effect of supplementation (*P* < 0.01) and grazing system (*P* < 0.05) without interaction. An overall decrease was observed for the weights of white offal (rumen, leaf, abomasum, small, and large intestine) and red offal (lungs, heart, and liver) with a significant effect of grazing system and supplementation without interaction. The proportions of prime cuts (shoulder, neck, leg, and ribs) in the carcass and shoulder components (muscle, bone, intermuscular fat, and muscle-to-bone ratio) did not change significantly.

**Table 3:** Carcass traits of Creole kids reared in rotationally grazed pasture according to the experimental groups.

**Pasture management**	**Control**		**Mixed grazing**			***P*****-value^b^**		
**S group^a^**	**Szero**	**Splus**	**Szero**	**Splus**	**SEM**	**PM**	**S**	**PM × S**
N^c^	6	6	6	6				
Slaughter weight (kg)	14.9	16.52	16.9	20.0	0.7	0.01	0.01	NS
EBW (kg)	11.9	13.8	13.1	16.5	0.6	0.01	0.01	NS
Hot carcass weight (kg)	5.8	6.9	6.5	8.5	0.3	0.01	0.01	NS
Cold carcass weight (kg)	5.5	6.7	6.1	8.1	0.3	0.01	0.01	NS
Dressing percentage (%)	45.9	48.1	46.5	49.1	4.6	NS	NS	NS
**Carcass scores**								
Conformation(1–5)	1.8	2.7	1.8	2.8		NS	0.01	NS
Internal fat(1–5)	1.5	1.8	2.8	3.2		0.05	0.01	NS
External fat(1–5)	2.8	2.8	2.7	2.9		NS	NS	NS

a *Szero: no supplementation, Splus: pellet 20 g/kg^–0.75^*.

b *Effect of pasture management (PM), supplementation (S), or interaction PM × S: level of significance; NS, non-significant*.

c *N, number of animals per group*.

## Discussion

The association of ruminant species with different grazing behaviors and level of susceptibility to GIN would allow the improvement of the efficiency of grazing ruminant production both by the improvement of the nutritional and the parasitological status. The mixed grazing system associating goats and cattle has been much less studied than sheep and cattle association, although the expected benefit to goat farming would be potentially important, given the greater susceptibility of goats to GIN infection (1). In this experiment, fodder production and chemical composition were within the high values reported previously (12). The biomass and its nitrogen concentration offered in the Mixed grazing groups were improved by 20 and 18.5% compared with the Control groups (Szero and Splus, respectively). These observations could be explained by the hypothesis of dietary complementarity between the different ruminant species (21). Indeed, it has been shown with the same breeds that goats consumed the upper parts of the canopy, while cattle consumed all the parts of the herbaceous canopy with the help of their very prehensile tongue, thus reducing the residual biomass (22). This more complete harvesting of forage biomass in mixed compared with single grazing system would be beneficial to subsequent regrowth, which would undoubtedly improve the proportion of foliage and, consequently, the nitrogen content of the forage. However, to support this hypothesis the measurement of the morphological structure of the forage on pasture (percentages of leaves, stems, and debris) to assess the quality of the forage offered on entry and the forage rejected on exit should have been done (23).

The evolution of the LW was within the average growth performance usually observed in Creole goats in post-weaning and significantly different between the experimental groups (19). In keeping with previous studies evaluating the effect of mixed grazing either concomitantly (as in the present study) or in a “leader” goat and “follower” cattle design with the same breeds, the supplementation improved the growth (20, 22). The evolution of weight and body condition, as a function of time, showed a hierarchy of these variables according to an increasing gradient of supplementation and association (from the non-supplemented Control group toward the supplemented Mixed grazing group).

This means that the FEC reflected the parasite pressure on the pasture. In accordance with previous studies, the average level of infection of the kids whatever the group is, exceeded a value of 800 for FEC as early as 65 days post-entrance at pasture (20, 24). The FEC increased to reach a peak at 100 days post-entrance at pasture, and as previously shown, a lower level of parasitism in the Mixed grazing system was observed (20, 25). Indeed, the number of kids for which the experiment was discontinued according to the PCV, together with FEC was higher in the Control groups. Furthermore, in the present study, no mortality was observed, while no targeted drenching was applied in the work of Mahieu showing rates of mortality ranging from 12.2 to 27.6%, and Marley et al. applied systematic drenching to all the groups. In keeping with the FEC, a significant effect of the mixed grazing system was also observed for the PCV since the most prevalent GIN in Guadeloupe is *Haemonchus contortus*, a hematophagous GIN species (26). The association of Creole kids and cattle positively impacted the PCV with a significant reduction in the induced anemia when compared with the Control groups. Under alternative grazing conditions between sheep and cattle, the same positive significant effect of the mixed grazing system was observed in different breeds of sheep and cattle (22, 27–29). For the PCV, there was a gain of eight points of kids of the Control groups supplemented, while it remains the same for the Mixed grazing groups, suggesting that supplementation reduced the deleterious impact of parasitism. Indeed, although kids of the Control groups showed the same level of parasitism measured through FEC, the supplementation improved significantly the production performances (i.e., BCS, LW, and carcass traits) of kids in this group. This effect of the dietary supplementation was also observed previously in studies showing up to 60% of reduction in the anemia induced by *H. contortus* infection in kids (30–32). These results suggest a close interaction between dietary supplementation and the level of parasitism.

In the tropics, goat farming is mainly oriented toward meat production (33). As demonstrated in previous studies on the slaughter performance of Creole goat, supplementation led to improved slaughter performance, carcass yields, and conformation scores (19, 34). Beyond, in these significant improvements of production performances here, we showed that supplementation could also improve the host protective response. Furthermore, the grazing system induced a variation in exposure to NGI, limiting the consequences of their deleterious effects on the carcass for kids in the Mixed grazing groups. There is little work related to this type of study. The same overall results were observed on parasitized local goats in Greece (35). Here, we combined two components of the integrated management of GIN parasitism in a goat farm: dietary supplementation and mixed grazing system. Interestingly, the advantage driven either by mixed grazing or feed supplementation on kids' performances would be equivalent.

## Data Availability Statement

The original contributions presented in the study are included in the article/supplementary materials, further inquiries can be directed to the corresponding author/s.

## Ethics Statement

The animal study was reviewed and approved by Comité d'Ethique en Matière d'Expérimentation Animale des Antilles et de la Guyane, C2EA-69.

## Author Contributions

GA, J-CB, and WC conceived and designed the experiments. WC, RA, and the Gardel team collected samples for the haematological and parasitological analysis. WC, VC, and BB performed the laboratory and the carcass analysis. WC, J-CB, GA, and NM performed the statistical analysis and wrote the paper. All authors read and approved the final manuscript.

## Conflict of Interest

The authors declare that the research was conducted in the absence of any commercial or financial relationships that could be construed as a potential conflict of interest.
